# Indicators on firm level innovation activities from web scraped data

**DOI:** 10.1016/j.dib.2022.108246

**Published:** 2022-05-06

**Authors:** Sajad Ashouri, Arho Suominen, Arash Hajikhani, Lukas Pukelis, Torben Schubert, Serdar Türkeli, Cees Van Beers, Scott Cunningham

**Affiliations:** a Quantitative Science and Technology Studies, VTT Technical Research Centre of Finland, Tekniikantie 21, 02044 Espoo, Finland; b Industrial Engineering and Management, Tampere University, Korkeakoulunkatu 8. PL 541, 33014 Tampereen Yliopisto, Finland; c Public Policy and Management Institute, Gedimino pr. 50 LT-01110 Vilnius, Lithuania; d Fraunhofer Institute for Systems and Innovation Research ISI, Breslauer Str. 48, 76135 Karlsruhe, Germany; e CIRCLE - Centre for Innovation Research, Sölvegatan 16, 22100 Lund, Sweden; f UNU-MERIT UM – United Nations University Maastricht Economic and Social Research Insitute on Innovation and Technology, Maastricht University, Boschstraat 24, 6211 Maastricht, the Netherlands; g Section Economics of Technology and Innovation, Department of Technology, Policy and Management, Delft University of Technology, Jaffalaan 5, the Netherlands; h School of Government and Public Policy, University of Strathclyde, Glasgow G1 1XQ, United Kingdom; i LUT University, School of Business and Management (LBM), Yliopistonkatu 34, 53850 Lappeenranta, Finland

**Keywords:** Big data, Web scraped data, Text data, Innovation, Firm-level data

## Abstract

This article presents data on companies' innovative behavior measured at the firm-level based on web scraped firm-level data derived from medium-high and high-technology companies in the European Union and the United Kingdom. The data are retrieved from individual company websites and contains in total data on 96,921 companies. The data provide information on various aspects of innovation, most significantly the research and development orientation of the company at the company and product level, the company's collaborative activities, company's products, and use of standards. In addition to the web scraped data, the dataset aggregates a variety firm-level indicators including patenting activities. In total, the dataset includes 21 variables with unique identifiers which enables connecting to other databases such as financial data.

**Specifications Table**

**Table:** 

Subject area	Management of Technology and Innovation
More specific subject area	Big data in innovation management
Type of data	Web scraped data; Text data
How data were acquired	Data were acquired by web scraping companies’ website.
Data Format	Semi-structured (raw and preprocessed)
Description of data collection	The relational database has stored web scrapped data of companies’ websites as a PostgreSQL database and stored on the virtual machine in the Microsft Azure Cloud. Some data tables are constructed by linking the web scrapped data to publicly available data.
Data source location	Sample of med-high and high-technology companies based in EU-27 and UK.
Data accessibility	Repository name: DataverseNL Data identification number: https://doi.org/10.34894/BS9XVR Direct link to the dataset: https://dataverse.nl/dataset.xhtml?persistentId=doi:10.34894/BS9XVR Also please follow the acknowledgment for more details.

**Value of the Data**

• The dataset extends previous work on firm-level innovation indicators by offering a novel vantage point provided by web scraped data.
• The dataset is useful for researchers who want to study innovation outcomes, economic complexity and collaboration through a large firm-level sample.
• The dataset informs on the process and potential of web scraping as a method to create innovation related micro-level data at scale.
• The dataset is beneficial to practitioners and policymakers for increasing awareness of the role of big data used for public policymaking.

## 1 Data Description

Much of the quantitative firm-level innovation research relies on structured data made available by either third-party organizations, surveys, or interviews. Datasets aim to capture innovation inputs (e.g. R&D expenditure) and to create practical indicators for measuring innovation outcomes (e.g. through patents). Datasets are also used to evaluate the impact of innovation at the micro- or macroeconomic level. The dataset presented in this paper offers a novel vantage point for firm-level innovation analysis by capturing innovation input, activity and output-related data collected from company websites. A further value of this dataset is the further transformation of this data according to theoretical concerns. Websites offer a rich source of information on company behavior [1], [2], [3], [4]. This information is stored in an unstructured heterogeneous format that requires significant pre-processing prior to creating variables. The information on the websites is provided as mixed visual and textual content designed for different communicative purposes [5]. These diverse purposes include marketing, human relations, and investor relations. The express purpose of websites is not exclusively to communicate technical expertise or innovative capability, nonetheless, previous work demonstrates the strong correlations of web texts with a variety of innovative measures including R&D expenditures, R&D employment, and patenting activity [6]. Exploring the textual content of websites has the potential to reveal a number of different facets of corporate activity, including the strategic orientation, the skills acquisition and innovation outcomes (product) of the company. Text mining approaches in innovation research enable the analysis of massive quantities of data delivered at scale [7]. With a rigorous analytical platform, large-scale quantitative analysis of text data enhances the transparency and reproducibility of results [8].

**Fig. 1:**
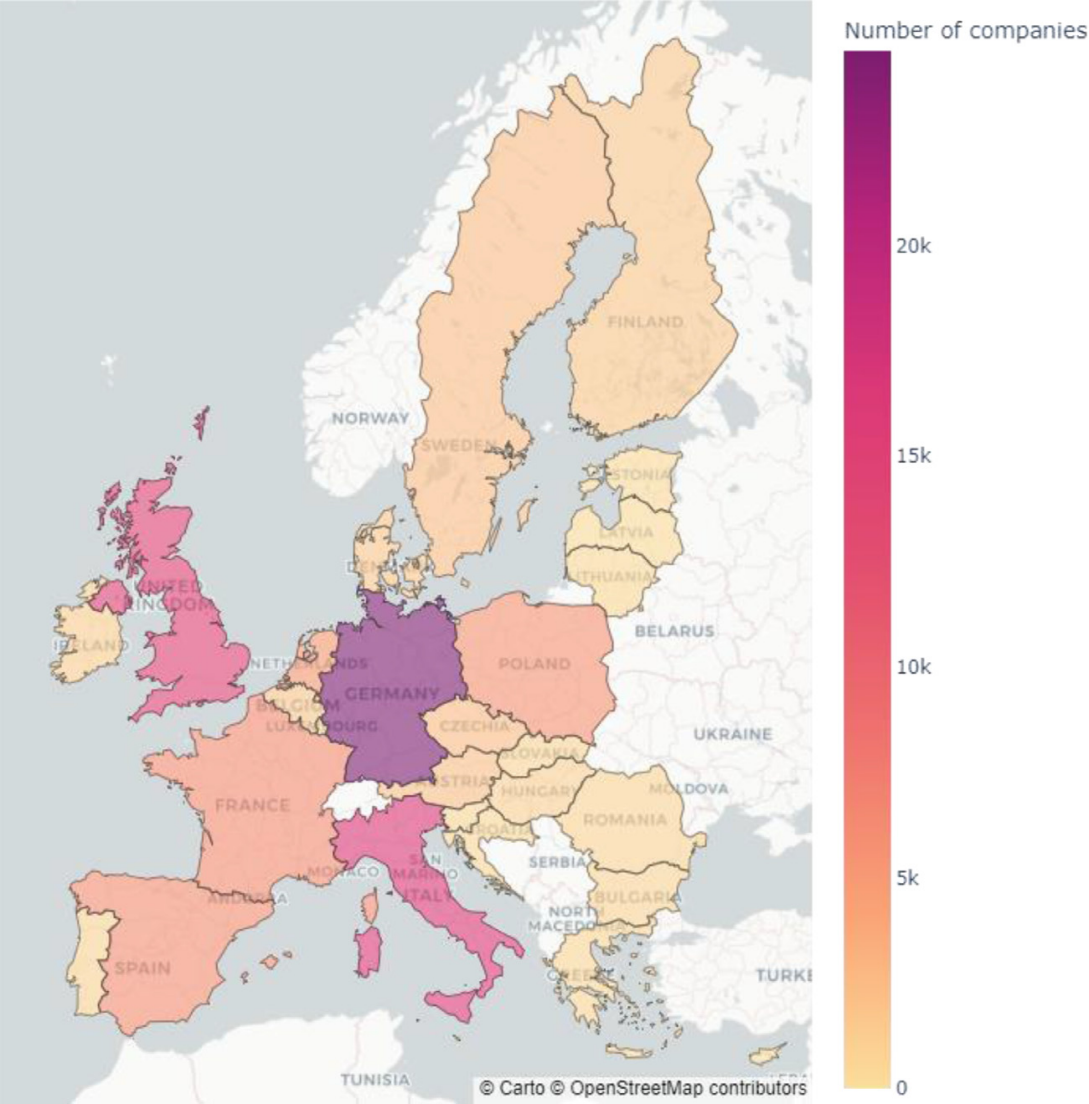
The heatmap showing the geographical distribution of companies in the dataset.

The datasets made available in this research are created by identified companies registered in the European Union and the United Kingdom, and by linking these addresses to corporate websites. The web scraper extracted the textual content of companies' websites, and thereafter transformed the data into innovation related variables and semi-structured data. The data is then stored on the platform for further analysis. The data has been gathered for a stratified sample of 183,161 companies in the medium-high and high-tech sectors from 27 European Union countries and the United Kingdom. The sample has been created using the Bureau van Dijk Orbis database, resulting ultimately in 96,921 distinct instances. Fig. 1 shows the distribution of companies across EU27 and UK. Germany, the United Kingdom, and Italy have the highest number of companies in our sample. Company websites for the process were taken from the Orbis database, and then complemented with a Google Search API to fill missing values concerning physical presence or address. Utilizing the company's URLs as a start URL, the automated website scrapping system traversed each page of the company's website to retrieve innovation related content. Cleaning, harmonizing, and tagging procedures are then applied to the text data. The retrieved data was thereafter connected to several third-party datasets such as public patent databases. In addition, Microsoft Academic Graph (MAG) is used as a taxonomy for knowledge used to reduce the dimensionality of the textual content.

While many subfields in innovation studies can benefit from such more flexible data, the literature in productivity analysis may have a particular urgent need for such novel types of data. A major reason for this urgency is that conceptual research argues that a number of structural changes to the innovation process are currently ongoing. This calls into question the applicability of traditional innovation and productivity indicators. Notably, [9] summarize the importance and increasing role of intangibles, servitization, and spillovers as a major driver of productivity and innovativity differentials between firms. Preliminary research along these lines demonstrates that AI-related capabilities can significantly boost firm-level productivity. However, this boost takes time to emerge and to be captured; and there is also a second-mover advantage [10]. Ongoing research in the project attempts to integrate the collected data into a CDM-type of model [11] in order to better obtain an integrated view of the joint productivity effects of the changes in innovation processes. Moreover, once repeated observations become available – for currently the dataset is based on single observations in time – there could be a means to define product and process innovations from firm-level data. The current state of the art relies on the definitions of the OSLO manual 2018 [12] and builds on survey data in isolation. Attempts to work starting from the foundational definitions of innovation are actually not new and have been implemented already in the SAPPHO and the SAPPHO II projects [13], and more recently in the SWINNO [14]. Although these approaches are accurate, they rely heavily on manual effort and are therefore too expensive to scale up to larger populations of firms and across wider settings. In that respect, the dataset and the methodology presented here also lays a foundation for fulfilling an almost 50 years journey towards a definition and measurement of innovation which extends standard survey techniques. This discussion of the applicability of the data provided here is in no sense fully exhaustive. The data to be fully described in the following sections can be applied to much broader topics including the role of digital technologies in innovation, the role and measurement science-industry links, the nature of firm internationalization, and the development of innovation alliances.

It is worth noting that the present data does not replace well-structured data sources, with value based on careful curation and a high degree of reliability. Instead, our data and resultant innovation indicators should be seen as a complementary data source. Such a source is relevant given the incentive of firms to accurately broadcast multiple aspects of their research and development activities to interested parties. Our approach is conceptually therefore a "bottom-up" rather than a scientifically construed "top-down" approach.

**Fig. 2:**
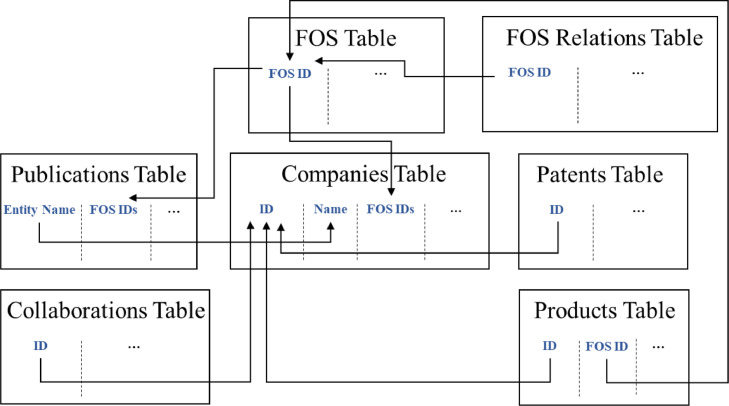
Relational database including seven data tables constructed from text data analytics.

The dataset is structured as a relational database, as can be seen in Fig. 2. The relational database consists of seven data tables, that contain different aspects of a company's innovation activities. Tables can be connected to one or more data tables based on unique identifiers. The Companies Table contains the basic information of the company and web scraped data at the company level. The Product Table contains a web scraped extraction of company products. Publication and Patent tables link the company to third-party scientific publications and patent information. The FOS (Field of Study) Table and the FOS Relations Table link company and product level information to the Microsoft Academic Graph. The Collaboration Table contains information on the collaborative partners mentioned on the company web page.

Table 1 describes each variable in the tables as seen in Fig. 2 with the data format, description, and source table the variable is stored. Table 2 shows the number of missing values for the datasets. Missing values occur when the source web page does not contain relevant data for that variable. For example, if a website does not communicate any information about standards, the ISO variable for the company will be classified using a missing value. Tables 3 and 4 show the descriptive statistics of the variables. The dataset includes only one numerical variable, namely the paper count given in the FOS table. Therefore, as can be seen in Table 3, descriptive statistics of the variables hold the number of unique instances as well as the most frequent values and their frequencies. In addition, Table 4 shows descriptive statistics for selected categorical variables from the Companies and Product tables. These statistics are created based on the length of vector, considering for example how many ISO codes a company is associated with.

**Table 1:** Description of the variables in each data table.

Variable	Data format	Description	Source table
ID	Varchar	Unique identifier of company	Companies Table
ISO	Dictionary including {ISO code: full name of ISO standard}	ISO standards associated with the company.	Companies Table
Keywords	Dictionary including {keyword: frequency in the text}	Meaningful keywords extracted from company's text.	Companies Table
FOS IDs	Dictionary including {fos_id: frequency in the text}	FOS id number and it's similarity score for the company. FOS names can be found in FOS table.	Companies Table
Linked countries	Dictionary including: {country_code: score}	Countries that are mentioned in the company's website text (not banners, ribbons or such). The scores are Min-Max scaled mention counts. e.g., score of 0.5 means that that country constitutes 50% of all country mentions.	Companies Table
Timestamp	timestamp	Timestamp of when the entity was uploaded to the database.	Companies Table
ID	Varchar	Unique identifier of company	Collaborations Table
Name	Varchar	Name of the collaborator	Collaborations Table
Category	Varchar	Country code of collaborator ("RPO/University" or "Other")	Collaborations Table
Country code	Varchar	Country code of collaborator	Collaborations Table
ID	Varchar	Unique identifier of a company	Patents Table
Patent ID	Varchar	Patent publication number	Patents Table
DOI	Varchar	Unique identifier of publication	Publications Table
FOS IDs	List	List of fos ids associated with the publication	Publications Table
ID	Varchar	The company associated with the publication	Publications Table
ID	Varchar	Unique identifier of a company	Products Table
Product Name	Varchar	Name of the Company	Products Table
Trademark	Boolean	If set True, the extracted product is a trademark	Products Table
Product FOS	Dictionary including {FOS id: Frequency}	List of FOS ids associated with the publication	Products Table
Product Keywords	List	List of keywords associated with the products	Products Table
FOS ID	Varchar	FOS identifier	FOS table
FOS name	Varchar	FOS name	FOS table
Level	Categorical	Indicates FOS level in hierarchical structure. Can be 1-5.	FOS table
Paper count	Numerical	Count of publications in Microsoft Academics database with specified FOS	FOS table
Parent ID	Varchar	Parent FOS ID	FOS Relations Table
Child ID	Varchar	Child FOS ID	FOS Relations Table

**Table 2:** Number of missing/not reported values for each variable.

Variable	Missing values/ not reported	Source table	Variable	Missing values/ not reported	Source table
ID	0	Companies Table	FOS IDs	0	Publications Table
ISO	61919	Companies Table	Similar Companies	0	Publications Table
Keywords	78	Companies Table	Bvd ID	0	Products Table
FOS IDs	4566	Companies Table	Product Name	0	Products Table
Linked countries	5614	Companies Table	Trademark	0	Products Table
Timestamp	13789	Companies Table	Product FOS	66299	Products Table
ID	0	Collaborations Table	Product Keywords	0	Products Table
Name	0	Collaborations Table	FOS ID	0	FOS Table
Category	0	Collaborations Table	FOS name	0	FOS Table
Country Code	35196	Collaborations Table	Level	0	FOS Table
Patent ID	0	Patents Table	Paper count	0	FOS Table
ID	0	Patents Table	Parent ID	0	FOS Relations Table
			Child ID	0	FOS Relations Table

**Table 3:** Summary statistics for categorical variables.

Variable	Source table	Unique observations	Top count
ID	Companies Table	96921	-
ISO code		3406	('ISO 9001′, 25861), ('ISO 14001′, 10749), ('ISO 13485′, 3398)
Linked Countries		195	('DE', 51943), ('US', 49695), ('IT', 34930)
FOS IDs		105527	('204441458′, 8628), ('2775945657′, 8593), ('160403385′, 7474)
Keywords		849203	('contact', 10261), ('product', 10169), ('high', 10145)

ID	Collaboration Table	18697	-
Name		57899	('FOOD AND DRUG ADMINISTRATION', 632), ('MINISTRY OF ECONOMIC AFFAIRS', 397), ('MINISTRY OF DEFENCE', 392)^1^
Category		2	Other
Country Code		190	('US', 55711), ('DE', 28376), ('GB', 25090)

ID	Patent Table	3114	-
Patent ID		361121	('US2015233026A1′, 25), ('CN105542514A ', 25), ('US2015217877A1′, 25)

FOS ID	Publication Table	69834	(71924100, 29685), (126322002, 18176), (41008148, 16493)
ID		3631	-

ID	Product Table	71082	('GB08774049′, 186), ('GBML3898974′, 176), ('GB02027512′, 150)
Product name		387420	('product portfolio', 468), ('management system', 386), ('surface finish', 377)
Trademark		2	('False’, 606892)
Product FOS		165763	('50549864′, 25201), ('122555611′, 18212), ('122707667′, 14822)
Product Keywords		109891	('type-members', 28312), ('pressure-testing', 19918), ('metal-insulator-semiconductors', 18909)

FOS ID	FOS Table	664968	-
FOS name		664968	-
Level		6	('3′, 321082), ('2′, 131604), ('4′, 111271)

Parent ID	FOS Relations Table	53933	('59822182′, 19563), ('141071460′, 9129), ('555293320′, 7993)
Child ID		429817	('2777753429′, 15), ('144623209′, 14), ('120592756′, 13)

1 Collaborators’’ names such as Food and Drug Administration do not record the country name in the name data column. To avoid the bias of such co-occurrences in future analyses, such collaborators' name should be recognized using their country code.

**Table 4:** Summary statistics for numerical variables.

Variable	Source table	Mean	Std	Min	25%	50%	75%	Max
#ISO codes	Companies Table	2.62	3.67	0	1	2	3	199
#Keywords		199.8	537.52	0	17	30	30	2000
#FOS IDs		88.82	25.65	0	100	100	100	100
#Linked countries		10.67	16.07	1	2	5	12	190

#Product keywords	Product Table	5.11	3.98	1	2	4	5	30
#Product FOS		13.37	27.87	1	3	3	8	474

Paper count	FOS Table	1873.3	76235.6	1	2	9	79	2807188

In this project, we also engineered a digitalization score from the available dataset. The digitalization score is a numerical variable which has been calculated for each of the products of a company; the average of scores across products provides the overall company product digitalization score. Table 5 indicates the descriptive statistics for the digitalization score.

**Table 5:** Descriptive statistics for the digitalization score.

Missing values	Mean	Std	Min	25%	50%	75%	Max
28948	0.095	0.202	0	0	0	0.100	1

**Fig. 3:**
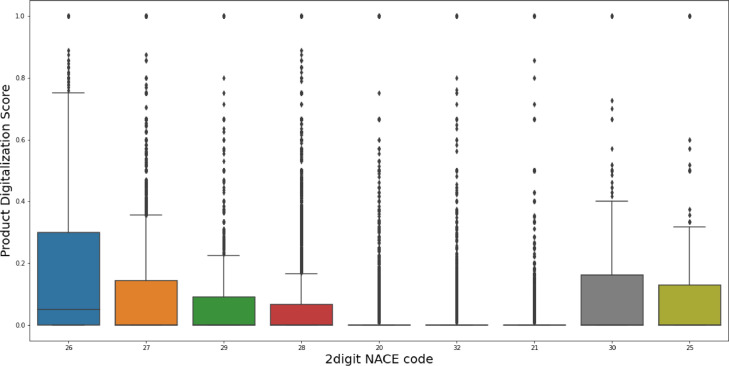
Distribution of product digitalization measure at the industry level (Nace code).

Figure 3 shows the distribution of product digitalization score at the industry level. As can be seen in this figure, the pharmaceutical and chemical industry, with NACE code 21 and 20 respectively, have the lowest product digitalization score. On the other side, the manufacture of computer, electronic and optical products obviously has the highest average for product digitalization score. However, the results of the measure are highly skewed in all the industries, which reflects the difference in focus on offering digital products within the industries.

## 2 Experimental Design, Materials and Methods

The following sections further elaborate on the data source identification, the process of data collection, and data integration to construct the database.

### 2.1 Data source identification and Data collection

• Identification of companies

The companies sample is selected to include companies with high potential for innovation activity. Thus, the primary focus is on measuring high-tech economic activity. The identification process uses the Eurostat aggregation of manufacturing industries based on its technological intensity and using the NACE revision 2 coding. NACE (Nomenclature of Economic Activities) is an industry standard classification system based on economic activities in the European community. The coding process utilized the NACE code at the 3-digit level aggregated for high-technology, medium high-technology, medium low-technology and low-technology groupings. The dataset is created by selecting companies that belong to the high-technology or medium-high-technology groups. These included the following NACE 3-digit codes and identifiers:

○ Manufacture of basic pharmaceutical products and pharmaceutical preparations (21)
○ Manufacture of computer, electronic and optical products (26)
○ Manufacture of air and spacecraft and related machinery (30.3)
○ Manufacture of chemicals and chemical products (20)
○ Manufacture of weapons and ammunition (25.4)
○ Manufacture of electrical equipment (27)
○ Manufacture of machinery and equipment n.e.c. (28)
○ Manufacture of motor vehicles, trailers and semi-trailers (29)
○ Manufacture of other transport equipment (30) excluding Building of ships and boats (30.1) and excluding Manufacture of air and spacecraft and related machinery (30.3)
○ Manufacture of medical and dental instruments and supplies (32.5)

Data collection is limited to companies that are currently active at the time of sample creation. To avoid constraining the sample excessively, companies with an unknown status were also included, but an additional requirement was given that the Orbis database needs to have website information for the company. Finally, the data has been created to be regionally bound to the European Union and the United Kingdom. This process resulted in 183,161 companies being included in the sample.

• Web scraping data platform

A data platform is specially designed to create a data retrieval, data harmonizing and data structuring pipeline to automate and facilitate the large-scale analysis of web data. The data platform used to web-scrape the sample companies utilizes a “hybrid” design with one part of the infrastructure being located on premises and the other part on a cloud-based platform (MS Azure Cloud Platform). The developed platform is composed of three main areas:

• Area 1: Main database (cloud)
• Area 2: Web-scraping and processing infrastructure (on-premises)
• Area 3: Public database and data exploration platform (cloud)

Area 1 is the main database that brings together company-level data from multiple sources, such as the Orbis database, indicators derived from unstructured company website data, indicators derived from semi-structured data sources (e.g. PATSTAT, European Union Intellectual Property Office (EUIPO)) and indicators derived from further analysis of the raw data. The collected data follows a uniform schema and is therefore implemented as a SQL (Postgre) database. The structure of the database can be seen in Fig. 1. The database is hosted in the Microsoft Azure Cloud Services. Area 2 is used to collect data from company websites and process and enrich it through the text-mining and text classification processes. Additionally, it facilitates linking data between the company website data and other data sources, such as PATSTAT for patents. As area 2 is involved in analyzing large amounts of text data, it is implemented as a NoSQL document database based on MongoDB. The database is populated by a multitude of worker processes that can interact with external sources and bring the data to the database. Another set of worker process ensures that the data relevant datasets in area 1 are kept up to date. In area 2, the data from the company, other websites as well as sources of semi-structured database data are pooled together. Then various data mining, information extraction, text classification and text fragment matching algorithms are run to:

• Identify and extract valuable pieces of information from the collected raw data
• Identify texts with relevant content for further detailed analysis
• Match fragments of text, e.g., product names to other records to link and enrich the data
• Construct indicators from the collected data.

The main element of this is a web scraper that uses the company website URL as a seed value to fully traverse the company website domain and extract text from pages and associated hyperlinks. Company websites are scraped using specially developed crawlers written in Python which recursively traverse the entire website and collect text data and links from each web page. The crawlers are set to collect only text data and to ignore images, PDFs, and other media files. As a considerable portion of the website content may be stored in the dynamically generated content pages upon visiting a new page on the company website, the scraping program captures the contents of the webpage and stores the data in a dedicated database. As a result, from the disaggregated dataset where a unit of analysis is a single URL representing the company as a whole, we build an over-arching and company-level dataset. This dataset contains all the raw data, and thereby the indicators which are most relevant to the analysis. The structured schema ensures interoperability across data sources.

In area 2 text mining is used to identify company products, the country names mentioned, standards used, and for the use of this project, collaborations mentioned on the website.

The final component of the platform is area 3, which facilitates access to the specified project datasets for end-users. We foresee that the main interest in the project results will come from specific groups:

• Scholars from academia;
• Policy professionals;
• Analysts from research organizations.

In other words, we foresee that, due to the highly technical nature of the project, the main groups interested in its results will be the people already working in the general sphere of business productivity.

### 2.2 Data processing and integration

  • Website text data cleaning

Texts from each individual webpage (URL) are stored separately and constitute the most granular unit of analysis. After the data collection, the website data undergo additional processing, which included removing boilerplate (sections of the website that occur in each URL, like headers, sidebars, or footers), language detection and machine translation to English. After such pre-processing, website data goes through additional processing steps to extract needed variables.

• Identification of FOS IDs using Microsoft Academic Graph

According to the high dimensionality and complexity of unstructured text data, we applied a novel approach of employing a large global publication database that can serve to measure the similarity between the structured data sources and text data. Microsoft Corporation has developed an open bibliometric database similar to Google Scholar, named Microsoft Academic^1^. Microsoft Academic Graph (MAG) is a large heterogeneous graph comprised of more than 200 million publications and the related authors, venues, organizations, and fields of study. As of today, the MAG is the largest publicly available dataset of scholarly publications and the largest dataset of open citation data [15]. Fields of Study (FOS) are the results of a hierarchical topic model run on the entire MAG data corpus. FOS IDs are introduced at five levels of detail, resulting in over 700,000 total topics and classifications.

Certain data elements like FOS fields are calculated with the data provided from MS Academic Graph. FOS data and the underlying keyword distributions for each FOS are referenced from a dump of MAG. This entails the complete download of the entire MAG dataset to a single user's storage account. We used the 2019-10-10 version of MAG dataset in this project. The methods on the website content transformation to FOS codes are explained extensivly in Hajikhani et al [16].

• Identification of products

One of the key steps in the data processing of this project is identifying and isolating product descriptions in the company website text. To this end, we employed a combination of machine learning (ML) and natural language processing (NLP) in the first stage of the process. ML models preselect website texts that are likely to contain product descriptions. Then a NLP model looks for certain phrase patterns in the texts (e.g. “we are glad to introduce our new…” + PROPER NOUN). These phrases help to isolate the product names in the website text data. Accordingly, the extracted data are processed to collect all the sentences from the company website that mention that particular product. These processes were applied for all companies' products and then, the data were stored as product data^2^.

• Identification of collaborations

We employed a similar approach for the identification of collaborations and entities, and used a combination of linguistic dependency parsing, entity recognition mechanisms, and machine-learning to identify collaborations. Using a set of phrase patterns and applying these patterns to the ML pre-selected texts, we increased the algorithm accuracy and minimized false positive instances. By using a list of pre-existing keywords regarding entity names as well as other specifications such as location, we also classified the entities into research and higher education sector entities and other organizational entities.

• Identification of standards

ISO standard codes were extracted using text mining techniques. The ISO code variable shows the ISO standards companies try to communicate with their audiences through their website. In the case that there is no ISO code identified on the website, this variable is reported as a missing value for the company. Importantly, the mention of ISO codes on the companies’ website does not guarantee that the company is actually applying or pursuing standards-setting process. Nonetheless, it does convey the awareness and concern of the company regarding particular standardization practices.

• Patent and Academic publications data

We also employed two other third-party databases to extract patents and academic publications for the corresponding entities studied in this project. To retrieve the patent data, we searched for the companies’ name through PATSTAT, and identified the relevant patents' name and their earliest filing date. Such data provide a proxy of firms’ innovation performance in high-tech industries [6]. To link the relevant academic publications, we also searched through the Microsoft Academic and identified the publications assigned to the companies’ name^3^.

• Product Digitalization score

This project employed a new methodology for measuring product digitalization, and translating digitalization into a single, and easily read score based on website texts. The process begins with specific high-level FOS identified in the Microsoft Academic Graph associated with computer science. Both the parent, as well as all children of these FOS codes, are associated with digitalization. Identified products on the company website are then recorded and associated with these FOS IDs based on a high level of shared or overlapped text. Computer science FOS are scored as one, non-computer science FOS are scored as zero. The aggregation and average of all products available on the website results in a ratio-scored variable ranging from zero to one (see Table 5 for the associated descriptive statistics of this variable). The new measure of product digitalization score, therefore, uses actual product description rather than conventional wisdom to determine whether a product is actually digital or not. An added value is the resultant aggregation of the entirety of the listed products of a company.

In conclusion, we believe the resultant data set will be valuable to a broad community of potential users, not the least of which are those researchers and policy-makers interested in firm-level productivity measures. The collected data provides information at a breadth and depth not previously seen in innovation studies. The resultant data drawing from exhaustive sources of open-source data is a very useful complement to highly structured and proprietary data sets. We furthermore believe that the process and architecture described herein will be useful to subsequent studies seeking to build upon and expand upon these findings.

## Ethics

**Terms of service (ToS):** Web crawling is an essential part of the functioning of the web. It is through this process that backlinks are essential to a website's visibility and searchability are generated. Hence, companies generally allow web crawling. The policy on which sections of the website can be scraped/crawled is usually outlined in a specific resource on the website called "robots.txt' file. During the web scraping process, we seek out ``robots.txt'' files, which indicate which sections of the website are accessible to crawlers. We therefore, obey the policy outlined there.

**Copyright:** This web scraping dataset only contains company websites. Neither social media nor news outlets are included. Furthermore, the data collected from company websites are not opened up or shared publicly. The shared data are only high-level indicators and processed data, which are constructed from the web textual contents as raw data.

**Privacy:** Since we deal with public data on companies, specifically the data that companies themselves opened up to the public. Therefore, this data do not need to be anonymized. Nonetheless, we consider some additional measures to avoid personal data or data falling under GDPR. We also adopt explicit measures to avoid capturing pages (like ``/contacts'') that might disclose contact information or any personal data.

**Scrapping policies:** Our scraping policy relies on locating and following directions in "robots.txt" files; and also, explicitly not collecting data from pages that might contain personal and contact information; Moreover, we avoid web scraping if a company employs any measures to limit/block crawlers (like rate-limits).

## CRedit Author Statements

**Sajad Ashouri:** Conceptualization, Methodology, Formal Analysis, Data curation, Investigation, Writing – original draft; **Arho Suominen:** Conceptualization, Methodology, Resources, Writing – original draft, Supervision, Project administration, Funding acquisition; **Arash Hajikhani:** Conceptualization, Methodology, Resources, Writing – original draft; **Lukas Pukelis:** Conceptualization, Methodology, Data curation, Resources, Investigation, Writing – original draft, Project administration, Funding acquisition; **Torben Schubert:** Conceptualization, Methodology, Writing- Original Draft, Supervision, Project Administration, Funding acquisition; **Serdar Türkeli:** Conceptualization, Methodology, Writing – original draft, Supervision, Project administration, Funding acquisition; **Cees Van Beers:** Conceptualization, Methodology, Writing – original draft, Supervision, Project Administration, Funding acquisition; **Scott Cunningham:** Conceptualization, Methodology, Writing – original draft, Supervision, Project administration, Funding acquisition.

## Declaration of Competing Interest

None. The authors declare that they have no known competing for financial interests or personal relationships which have, or could be perceived to have, influenced the work reported in this article.

## Data Availability

Replication Data for: BIGPROD Data Sample (Original data) (Dataverse).

## Footnotes

1 Source: https://academic.microsoft.com/home

2 We acknowledge that as the web scraper ignore the PDF files data, the products may have been mentioned in such files, would be missed.

3 We also acknowledge that identification of patents and publications which are registered with different name, however they are developed by the same company, would be missed in our records.
